# Glutathione Homeostasis and Functions: Potential Targets for Medical Interventions

**DOI:** 10.1155/2012/736837

**Published:** 2012-02-28

**Authors:** Volodymyr I. Lushchak

**Affiliations:** Department of Biochemistry and Biotechnology, Vassyl Stefanyk Precarpathian National University, 57 Shevchenko Street, Ivano-Frankivsk 76025, Ukraine

## Abstract

Glutathione (GSH) is a tripeptide, which has many biological roles including protection against reactive oxygen and nitrogen species. The primary goal of this paper is to characterize the principal mechanisms of the protective role of GSH against reactive species and electrophiles. The ancillary goals are to provide up-to-date knowledge of GSH biosynthesis, hydrolysis, and utilization; intracellular compartmentalization and interorgan transfer; elimination of endogenously produced toxicants; involvement in metal homeostasis; glutathione-related enzymes and their regulation; glutathionylation of sulfhydryls. Individual sections are devoted to the relationships between GSH homeostasis and pathologies as well as to developed research tools and pharmacological approaches to manipulating GSH levels. Special attention is paid to compounds mainly of a natural origin (phytochemicals) which affect GSH-related processes. The paper provides starting points for development of novel tools and provides a hypothesis for investigation of the physiology and biochemistry of glutathione with a focus on human and animal health.

## 1. Introduction

 Glutathione (GSH) is a tripeptide (L-*γ*-glutamyl-L-cysteinyl-glycine) with multiple functions in living organisms [1–4]. As a carrier of an active thiol group in the form of a cysteine residue, it acts as an antioxidant either directly by interacting with reactive oxygen/nitrogen species (ROS and RNS, resp.) and electrophiles or by operating as a cofactor for various enzymes [5–8]. Glutathione is moderately stable in the intracellular milieus because intracellular peptidases can cleave peptide bonds formed by the *α*-carboxyl groups of amino acids, but typically not the *γ*-carboxyl groups.

 The reduced and oxidized forms of glutathione (GSH and GSSG) act in concert with other redox-active compounds (e.g., NAD(P)H) to regulate and maintain cellular redox status [9]. The former is quantitatively described by the redox potential, calculated according to the Nernst equation. In most cells and tissues, the estimated redox potential for the GSH/GSSG couple ranges from −260 mV to −150 mV (cited after [10]).

 GSH is synthesized in a two-step process catalyzed by L-glutamate: L-cysteine *γ*-ligase, (*γ*GLCL, EC 6.3.2.2) (also called *γ*-glutamyl-L-cysteine ligase or *γ*-glutamylcysteine synthase), and glutathione synthase (GLS, EC 6.3.2.3). GSH is consumed in many ways, such as by oxidation, conjugation, and hydrolysis [11]. GSH can be directly oxidized by ROS and RNS or indirectly during GSH-dependent peroxidase-catalyzed reactions. Conjugation with endogenous and exogenous electrophiles consumes a substantial portion of cellular GSH. In addition, cells may lose GSH due to export of its reduced, oxidized or conjugated forms. Extracellularly, GSH can be hydrolyzed by *γ*-L-glutamyl transpeptidase (GGT, EC 2.3.2.2) transferring the *γ*-glutamyl functional group to water during hydrolysis to form free glutamate [12]. The enzyme may also transfer the *γ*-glutamyl moiety of GSH to amino acids and peptides. Frequently, products of GSH hydrolysis are taken up by cells either as individual amino acids, or as dipeptides. The intra- and extracellular GSH levels are determined by the balance between its production, consumption, and transportation. Due to important physiological functions of GSH, these processes are tightly regulated. The activities of the enzymes involved in GSH metabolism are controlled at transcriptional, translational, and posttranslational levels [3, 11].

 Since GSH participates not only in antioxidant defense systems, but also in many metabolic processes, its role cannot be overestimated. Therefore, it is not surprising that the GSH system has attracted the attention of pharmacologists as a possible target for medical interventions. The main efforts in this field have been applied to decreasing or increasing GSH levels in organisms. General strategies involve specific inhibition of *γ*GLCL, a key enzyme of GSH biosynthesis, and depletion of cellular reserves by externally added electrophiles (usually for research purposes). The use of buthionine sulfoximine (BSO) is probably the most popular approach to depleting GSH. BSO was first synthesised as the D,L-form [13, 14] and later as the L-BSO enantiomer [15]. Usually a mixture of D- and L-BSO is used in experiments [16–18]. GSH levels may be enhanced by supplementation with precursors, mainly cysteine in the form of different esters. However, during the the last decade a new approach for the regulation of GSH-utilizing enzymes has emerged. It is evident that many of these are induced at the transcriptional level by mild oxidative stress, which involves binding of the Nrf2 transcription factor to the antioxidant response element (ARE) (also called the electrophile response element; EpRE) in the promoter region of genes encoding certain enzymes, particularly *γ*GLCL and glutathione *S*-transferases [19–22].

 Glutathione has several additional functions in cells. For example, it is (i) a reserve form of cysteine, (ii) stores and transports nitric oxide, (iii) participates in the metabolism of estrogens, leukotrienes, and prostaglandins, the reduction of ribonucleotides to deoxyribonucleotides, the maturation of iron-sulfur clusters of diverse proteins, (iv) involved in the operation of certain transcription factors (particularly those involved in redox signalling), and (v) the detoxification of many endogenous compounds and xenobiotics [11].

 The present review will focus on the molecular mechanisms of operation of the GSH system, with special attention to regulatory pathways controlling the expression of the enzymes involved. Information on GSH biosynthesis, hydrolysis and utilization, intracellular compartmentalization, and interorgan transfer will be highlighted. Special sections will deal with GSH functions, such as antioxidant properties and relationship to specific enzymes. On the basis of these mechanisms, some potential approaches for medical interventions will also be evaluated.

## 2. Glutathione Biosynthesis, Hydrolysis, Excretion, and Utilization

Intracellular GSH concentrations usually range from 0.5 to 10 mM, whereas extracellular values in animals are one to three orders of magnitude lower [2, 11]. GSH is commonly the most abundant low molecular mass thiol in animal and plant cells. Most microorganisms also possess GSH in high concentrations, but there are some species and viable mutants lacking GSH [23–25].

**Figure 1:**
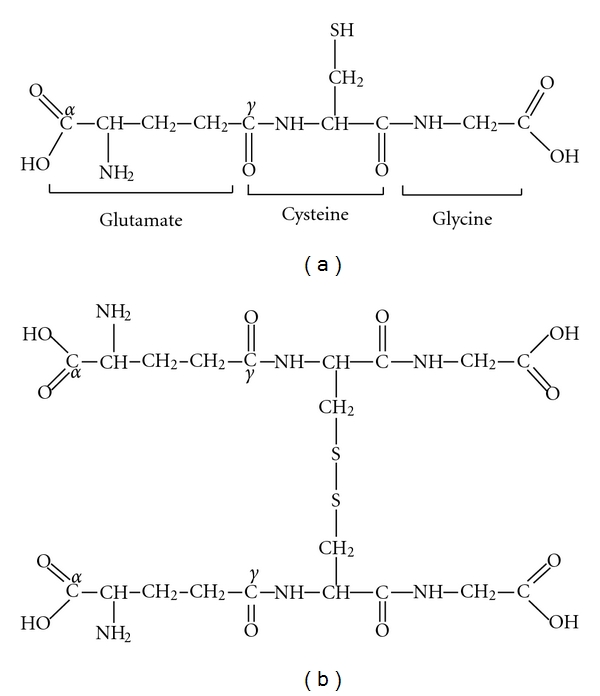
Glutathione is a tripeptide: L-*γ*-glutamyl-L-cysteinyl-glycine. In its reduced form (a) the N-terminal glutamate and cysteine are linked by the *γ*-carboxyl group of glutamate, preventing cleavage by common cellular peptidases and restricting cleavage to *γ*-glutamyltranspeptidase. The cysteine residue is the key functional component of glutathione, providing a reactive thiol group that plays an essential role in its functions. Furthermore, cysteine residues form the intermolecular dipeptide bond in the oxidized glutathione molecule (b).

Figure 1 shows the chemical structure of reduced and oxidised glutathione forms. GSH is formed from glutamate, cysteine, and glycine (Figure 1(a)), but it possesses an unusual peptide bond. The N-terminal glutamate and cysteine residues are linked by the *γ*-carboxyl group of glutamate, rather than the common linkage in proteins of an *α*-carboxyl peptide bond. This specific peptide bond prevents GSH from being hydrolyzed by most peptidases that cleave at the *α*-carboxyl peptide bond of N-terminal amino acids. This configuration also restricts the cleavage of GSH by GGT localized on the external surface of certain cell types. As a result, GSH is relatively stable in the cell and is cleaved by GGT only at external sides on the membranes of certain cells. In addition, the presence of the C-terminal glycine residue in the GSH molecule protects it against cleavage by intracellular *γ*-glutamyl cyclotransferase. The major oxidized form of glutathione (i.e., glutathione disulfide, GSSG) consists of two residues of GSH that have been oxidized in such a fashion as to be connected by an intermolecular disulfide bond (Figure 1(b)).

**Figure 2:**
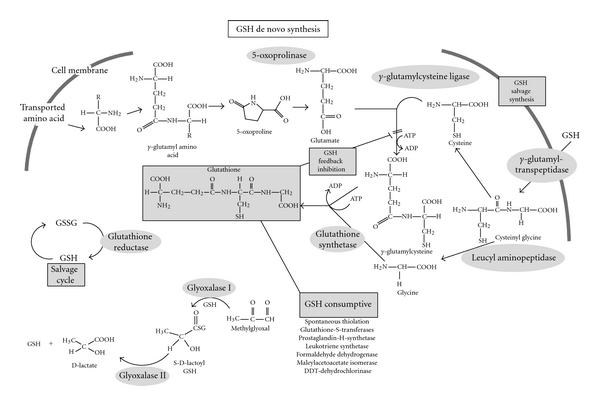
Glutathione homeostasis involves both intra- and extracellular mechanisms. Glutathione is synthesized in both *de novo* and salvage synthesis pathways. *De novo* synthesis requires the three amino acids and energy in the form of ATP. Glutamate may be provided in part from the conversion of a *γ*-glutamyl amino acid to 5-oxoproline, which is then converted to glutamate. Two ATP molecules are used for the biosynthesis of one GSH molecule. Salvage synthesis involves either reduction of GSSG or uses precursors formed from the hydrolysis of GSH or its conjugates by *γ*-L-glutamyl transpeptidase at the external surface of the plasma membrane that are transported back into the cell as amino acids or dipeptides. GSH is consumed in various processes. In addition to detoxification of reactive species and electrophiles such as methylglyoxal, GSH is involved in protein glutathionylation and several other processes, such as the biosynthesis of leukotrienes and prostaglandins, and reduction of ribonucleotides. Modified from [27].

The steady-state level of cellular GSH is provided by the balance between production and consumption, as well as by extrusion from the cell as reduced, oxidized, or bound forms (summarized in Figure 2). GSH is produced in two steps. In the first step, the enzyme *γ*GLCL forms a peptide bond between the *γ*-carboxyl of glutamate and the amino group of cysteine using energy provided by the hydrolysis of ATP:

(1) $$\;\begin{array}{cc} & \gamma \text{-L-Glutamate}\;+\;\text{L-cysteine}\;+\;\text{ATP}\\ & \;\to \;\gamma \text{-L-glutamyl-L-cysteine}+\;\text{ADP}\;+\;{\text{P}}_{\text{i}} \end{array}$$

In the next step, the dipeptide is combined with glycine by glutathione synthetase (GLS), again driven by the hydrolysis of ATP:

(2) $$\begin{array}{cc} & \gamma \text{-L-Glutamyl-L-cysteine}\;+\;\text{glycine}\;+\;\text{ATP}\\ & \;\to \;\text{GSH}\;+\;\text{ADP}\;+\;{\text{P}}_{\text{i}} \end{array}\;$$

It should be noted that, in some cases, the provision of ATP for GSH synthesis can be a limiting factor for GSH metabolism [26]. The first step, catalyzed by *γ*GLCL, is the rate-limiting step for overall GSH biosynthesis process. The enzyme is inhibited by GSH, the end product of the pathway, indicating that its biosynthesis is regulated via a negative feedback control mechanism.

**Figure 3:**
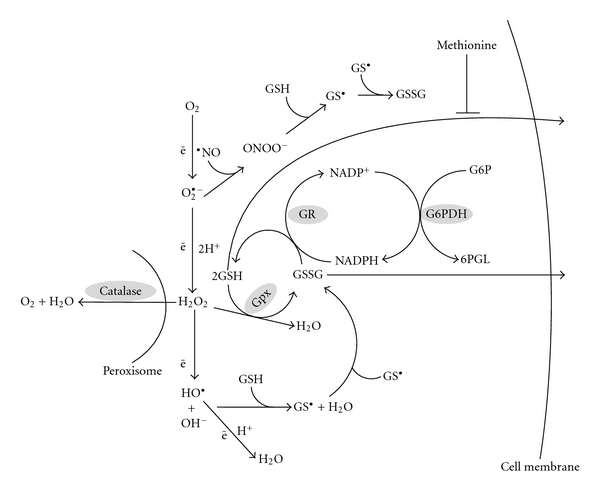
Involvement of glutathione in elimination of reactive oxygen and nitrogen species. Hydroxyl radical and nitric oxide (after oxidation to the NO^+^ form) or peroxynitrite may interact directly with GSH leading to GSSG formation. Hydrogen peroxide may be removed by catalase or by glutathione peroxidase (GPx). The latter requires GSH to reduce peroxide.

GSH may be oxidized directly by oxidants such as hydroxyl radical (HO^•^) [28, 29] or peroxynitrite (ONOO^−^) [30, 31]. Direct oxidation leads to the production of thiyl radicals [32], the fusion of which results in GSSG formation (Figure 2). GSH is extensively used as a cosubstrate by glutathione peroxidases (GPx, EC 1.11.1.9) reducing hydrogen peroxide (H_2_O_2_) or organic peroxides (generally abbreviated as ROOH or LOOH in the case of lipid peroxides) with the production of GSSG, water, or alcohols.  Figure 3 shows the dismutation of H_2_O_2_ by catalase.

How do catalases and GPxs cooperate in H_2_O_2_ catabolism? Firstly, they are mainly localized in different cellular compartments—GPxs are cytosolic residents, whereas catalases are found mainly in peroxisomes. Secondly, the affinity of GPx for H_2_O_2_ is one to two orders of magnitude higher than that of catalase. So, one may conclude that the two enzymes operate in concert, complementing each other. GSSG produced from the consumption of GSH can be either restored again by the action of glutathione reductase (GR, EC 1.6.4.2) (reaction (3)), or excreted from the cell.

(3) $$\text{GSSG}\;+\;\text{NADPH}\;+\;{\text{H}}^{+}\to 2\text{GSH}\;+\;{\text{NADP}}^{+}$$

Glutathione excretion from cells is inhibited by methionine [33]. Three forms of glutathione, namely, GSH, GSSG, and GSH-conjugates, can be excreted into extracellular spaces. There the conjugates are mainly hydrolysed to different components and reabsorbed. However, cysteine residues usually remain conjugated to xenobiotics and are released by organisms in feces. Most glutathione *S*-conjugates are metabolized to the corresponding *N*-acetyl cysteine *S*-conjugates (mercapturic acids) and released in the urine and bile [34]. Glutamate and glycine residues are usually recovered, but the cysteine residues remain conjugated and are lost. Both GSH and GSSG are substrates for the extracellular membrane-bound enzyme GGT:

(4) $$\begin{array}{cc} \text{GSH}+\;\text{amino}\;\text{acid} & \to \gamma \text{-glutamyl-amino}\;\text{acid}\\ & \;\;+\;\text{L-cysteinyl-glycine} \end{array}\;$$

(5) $$\text{GSH}\;+\;{\text{H}}_{2}\text{O}\;\to \;\text{L-glutamate}\;+\;\text{L-cysteinyl-glycine}$$

*γ*-L-Glutamyl transpeptidase cleaves only the *γ*-peptide linkage. The enzyme can transfer the *γ*-glutamyl group of GSH, GSSG, or GSH-conjugates onto amino acid acceptors to form *γ*-glutamyl peptides and cysteinylglycine (reaction (4)), or to water thereby hydrolyzing GSH and related compounds to glutamate and cysteinylglycine (reaction (5)). Cysteinylglycine can be further hydrolyzed by a dipeptidase to cysteine and glycine. The products, namely, amino acids and *γ*-glutamyl amino acids, may be transported back into cells and used for GSH resynthesis or other needs. This provides the basis for recycling of excreted GSH and GSSG (salvage cycle) by the cell of origin or by other cells [34]. Upregulation of this process provides an additional mechanism for GSH maintenance in the cell.

## 3. Intracellular Compartmentalization and Interorgan Transfer

Although GSH is synthesized in the cytosol, it is distributed to different intracellular organelles where it is used in organelle-specific functions related to its role in the regulation of cellular redox status. In addition to the cytosolic pool, GSH functions in somewhat independent pools in the endoplasmic reticulum (ER), nucleus, and mitochondria. In most of these compartments GSH is typically found in a highly reduced state, but in the ER a substantial portion is oxidised and the ratio [GSH]/[GSSG] may be as high as 3 : 1, whereas in the cytoplasm the oxidized form is usually on the order of about 1% of the total or less [35, 36]. In the ER, GSSG is the main source of oxidizing power that supports the efficient production of the functional conformation of nascent polypeptides by the formation of the required intramolecular disulfide bonds between cysteine residues. In the nucleus, GSH maintains the appropriate redox status of the sulfhydryl groups in proteins involved in nucleic acid biosynthesis and DNA repair in addition to standard antioxidant functions. In this compartment, it is also used in the reduction of ribonucleotides to produce deoxyribonucleotides by ribonucleotide reductase [37].

About 10–15% of cellular GSH is located in mitochondria. Since mitochondria have a very small volume, the local GSH concentration in these organelles is usually higher than that in the cytosol. Of the various subcellular compartments, most attention has been paid to the mitochondrial GSH pool (mGSH) because of the close relationship between mGSH and cell survival that has been demonstrated in many cases. This topic is covered in an excellent recent review of Mari et al. [38] and readers are directed to this review for extensive details. Here, I will mention just a few important aspects of the mGSH system. As mentioned above, GSH is synthesized only in the cytosol and is transported into intracellular organelles. It easily crosses the outer mitochondrial membrane through porin channels but, being an anion, cannot diffuse across inner mitochondrial membrane into the matrix. At least two systems are believed to be involved in GSH import into the mitochondria across the inner membrane. GSH transport into the matrix must overcome an unfavourable electrochemical gradient [39–44]. This is provided by two mitochondrial membrane carriers [45, 46] that exchange GSH for dicarboxylates and 2-oxoglutarate (*α*-ketoglutarate). These two antiport carriers provide electroneutral exchange of selected anions across the inner mitochondrial membrane with no charge transfer. The role of these two mitochondrial GSH carriers was also evidenced by a reconstitution of recombinant mitochondrial dicarboxylate carriers into proteoliposomes [45]. However, it should be noted that during GSH import the mitochondria lose important intermediates of the Krebs cycle so that anaplerotic mechanisms may be needed to replenish these. It should also be noted that GSSG cannot leave the mitochondria and therefore needs to be regenerated in the matrix by GR using NADPH (reaction (3)).

 In addition to its “classic” functions, GSH plays organelle-specific roles in the mitochondria and a few of them will be mentioned here. Due to the pivotal role of mitochondria in programmed cell death (apoptosis) as well as extensive ROS involvement in this process, and adding the fact that mitochondria produce over 90% of cellular ROS, the role of GSH in cell protection cannot be overestimated. GSH may either directly bind some ROS species or serve as a source of reductive power for certain antioxidant systems. The inner mitochondrial membrane is particularly rich in cardiolipin, whereas it is virtually absent from other membranes and only the outer mitochondrial membrane contains minor amounts of this phospholipid. When mGSH levels are compromised, cardiolipin is one of the important targets of oxidative damage. Due to its unique chemical structure among phospholipids, cardiolipin confers stability and fluidity to the mitochondrial membrane. In addition, cytochrome *c* is normally bound to the inner mitochondrial membrane via its association with cardiolipin. By protecting cardiolipin from oxidative damage, GSH prevents changes in the physicochemical properties of the mitochondrial inner membrane that lead to membrane destabilization and the dissociation of cytochrome *c*. ROS also induce an increase in permeability of the internal mitochondrial membrane for calcium. Enhanced ROS and calcium levels, acting in concert, may trigger the cell death machinery via apoptosis or necrosis. Hence, mitochondrial GSH clearly has an important role in preventing apoptosis triggered by cytochrome *c* release from the inner membrane.

 Not surprisingly, therefore, a decrease in mGSH levels is closely associated with certain pathologies in both humans and animals. This relationship has been described for hypoxia/reperfusion injury [47, 48], certain liver diseases such as alcoholic steatohepatitis [49, 50], nonalcoholic steatohepatitis [51, 52], and liver cirrhosis [53, 54], neurological diseases such as Alzheimer and Parkinson diseases, diabetes mellitus and associated complications [55–57]. Many of the abovementioned pathologies are included in the group of so-called age-related diseases and, therefore, it is not easy to differentiate aging as a normal physiological process and age-related or age-induced pathologies. Harman [58] proposed the oxidative stress theory of aging, which he later modified to the mitochondrial theory of aging [59]. This theory suggested that oxidative damage to organisms is connected with the progressive accumulation of oxidized/modified products of ROS attack that ultimately determine the lifespan of organisms. Insofar as they are cornerstones of the oxidative stress and/or mitochondrial theories of aging, ROS and mitochondrial function are intimately regulated by GSH and the [GSSG]/[GSH] ratio, thereby linking these theories of aging to mitochondrial GSH levels. Other pathologies, such as several diseases of the lungs (e.g., chronic pulmonary disease, acute respiratory distress syndrome, neonatal lung damage, and asthma) and of the immune system are also associated with a compromised mitochondrial GSH system [60–62]. Finally, mGSH involvement in combating the toxicity of different xenobiotics, particularly drugs such as cisplatin, is clearly evident [63–65].

One more important point related to mGSH should also be mentioned here. The correct analysis of the mitochondrial GSH pool is an experimentally complicated issue. To study this, cells are typically disrupted in order to isolate mitochondria and this can substantially affect not only redox status, but also total GSH content. Hence, there is a need to introduce new techniques for the proper evaluation of the operation of the mitochondrial GSH system. Some interesting ideas on this topic can be found in recent studies by Winther and colleagues [66, 67].

Another important topic is GSH distribution between different organs of animals. Glutathione can be transported across the plasma membrane, which is the first step of a complicated interorgan transfer network [4, 13]. Liver is the main source of GSH exported into the blood [68–71]. The export of GSH and its conjugates from liver cells occurs via transporters referred to as organic anion-transporting polypeptides (OATPs), which are generally believed to carry out electroneutral exchange, in which the cellular uptake of organic anions is coupled to the efflux of anions such as HCO_3_^−^, GSH, GSSG, and/or glutathione *S*-conjugates [72, 73]. Both GSH and GSSG are circulated and are used to supply other organs, particularly kidney. The production in liver and export from it are related to GSH functions, and at least two principles may be implicated. The first one involves epithelial cells that contact with the exterior, such as intestine and lungs. The primary GSH function here is directed to detoxification of injurious external agents to prevent damage to the organism. There is a large body of data indicating that this is an important role of GSH in normal intestinal function. The lungs are exposed to high oxygen levels and also to inhaled toxins. Alveolar macrophages provide an additional ROS source in this tissue. Hence, there are multiple reasons for maintaining adequate GSH levels in lungs. The second principle is related to high intensity oxygen-based metabolism and detoxification of certain compounds by internal organs. Liver and kidney are probably the best representatives of this group. The portal vein brings blood from the intestine to the liver and, if not detoxified in the intestine, xenobiotics must be neutralized by hepatocytes [52, 74–76]. In addition, the liver is an important biosynthetic organ where ROS are produced in substantial amounts as side products of energy production in the mitochondrial electron transport chain or as the result of biosyntheses involving diverse oxygenases. Kidney also requires a highly efficient GSH system to perform its functions [13, 77, 78]. The problems with extracellular GSH investigation and intertissue transfer are to a large extent based on inadequate methodology. Since the concentrations of extracellular GSH are more than an order of magnitude lower than intracellular levels, correct redox ratios are often difficult to determine.

## 4. Glutathione Functions

 The chemical structure of GSH determines its potential functions and its broad distribution among all living organisms reflects its important biological role. GSH has been found in all mammalian cells. Probably most importantly, GSH is responsible for protection against ROS and RNS, and detoxification of endogenous and exogenous toxins of an electrophilic nature. Other functions include (i) maintaining the essential thiol status of proteins and other molecules; (ii) storage of cysteine reserves both in the cell and for interorgan transfer; (iii) involvement in the metabolism of estrogens, leukotrienes, and prostaglandins; (iv) participation in the reduction of ribonucleotides to deoxyribonucleotides; (v) participation in the maturation of iron-sulfur clusters in proteins; (vi) copper and iron transfer; (vii) signal transduction from the environment to cellular transcription machinery. The above-listed GSH functions and a few others will be covered in this section.

### 4.1. Elimination of Reactive Oxygen and Nitrogen Species

GSH is an important antioxidant, directly reacting with ROS, RNS, and other reactive species, particularly HO^•^, HOCl, RO^•^, RO_2_^•^, ^1^O_2_, and  ONOO^−^, often resulting in the formation of thiyl radicals (GS^•^) (Figure 3). GSH is also involved as an antioxidant in the detoxification of products from ROS-promoted oxidation of lipids such as malonic dialdehyde and 4-hydroxy-2-nonenal [79, 80], and probably many other products of ROS interaction with cellular components [11, 19, 81, 82]. The thiyl radicals formed from these reactions can also combine with different molecules, as well as with other thiyl radicals leading to the formation of oxidized glutathione (glutathione disulfide, GSSG) in the latter instance. GSSG is also produced in reactions catalyzed by GPx (reaction (6)) and glutaredoxins (reaction (7)):

(6) $$\begin{array}{c} \text{ROOH}+2\text{GSH}\to \text{ROH}+\text{GSSG}+{\text{H}}_{2}\text{O} \end{array}$$

(7) $$\begin{array}{cc} & \text{Oxidized}\;\text{glutaredoxin}+2\text{GSH}\;\;\\ & \;\to \text{reduced}\;\text{glutaredoxin}+\text{GSSG} \end{array}$$

GSSG may be either excreted from the cell, or reduced by GR at the expense of NADPH (reaction (3)). Most of the reductive power for this reaction is provided by the pentose phosphate shunt-two molecules of NADPH are produced per molecule of glucose-6-P that cycle through the pathway. The first and limiting step is catalyzed by glucose-6-phosphate dehydrogenase (G6PDH, EC 1.1.1.49):

(8) $$\begin{array}{cc} & \text{Glucose-}6\text{-phosphate}+{\text{NADP}}^{+}\;\;\;\\ & \;\to 6\text{-phosphoglucolactone}+\text{NADPH}+{\text{H}}^{+} \end{array}$$

The second molecule of NADPH is provided by the next pentose phosphate shunt reaction, catalyzed by 6-phosphogluconate dehydrogenase (6-PGDH). These two enzymes are not the only cellular NADPH producers. NADPH is also formed by NADP-dependent isocitrate dehydrogenase, malic enzyme, and some others, but it is widely believed that most cellular NADPH is generated by the pentose phosphate pathway.

 As mentioned above, the glutathione couple GSH/GSSG is a critically important redox player and together with other redox active couples, including NAD(P)/NAD(P)H, FAD/FADH_2_, regulates and maintains cellular redox status. The estimated *in vivo* redox potential for the GSH/GSSG couple ranges from −260 mV to −150 mV depending on the conditions (cited after [10]).

 Under normal conditions, when a cell is not stressed, the processes that generate ROS are well counterbalanced by antioxidant systems. In this respect, GSH is often considered to be a key player of the defense system. However, under various circumstances the steady-state ROS level increases leading to oxidative damage to the cell, called “oxidative stress,” the term first defined by Sies [83] “*Oxidative stress” “came to denote a disturbance in the prooxidant-antioxidant balance in favor of the former*.” The definition was later expanded to “*An imbalance between oxidants and antioxidants in favour of the oxidants, potentially leading to damage, is termed “oxidative stress””* to emphasize the damage to certain cellular components [84]. Owing to extensive studies on oxidative stress and the discovery of many intricacies related to this phenomenon over the two last decades, the definition could be modified to “*Oxidative stress is a situation where the steady-state ROS concentration is transiently or chronically enhanced, disturbing cellular metabolism and its regulation and damaging cellular constituents*” [81]. This definition underlines the dynamic nature of the processes of ROS generation and elimination, damage to cellular core and regulatory pathways, and potential negative consequences of enhanced ROS levels either acutely or chronically. If cells are not capable of coping with the intensity of oxidative stress, this can culminate in their death via necrosis or apoptosis.

**Figure 4:**
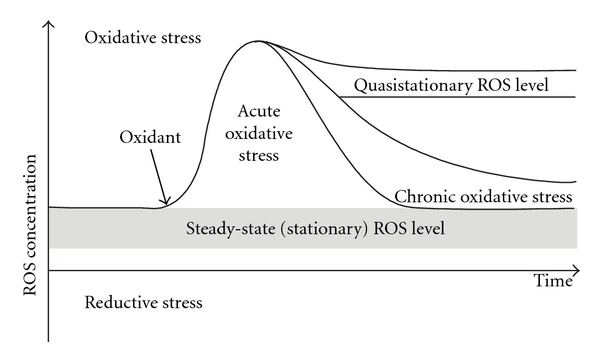
The dynamics of reactive oxygen species in biological systems. Steady-state levels of reactive oxygen species fluctuate over a certain range under normal conditions. However, under stress ROS levels may increase or decrease beyond the normal range resulting in acute or chronic oxidative or reductive stress. Under some conditions, ROS levels may not return to their initial range and stabilize at a new quasistationary level.

 The dynamics of ROS-related processes are shown in Figure 4. Under control conditions, steady-state ROS levels fluctuate over a certain range [81, 82, 85]. However, ROS levels can exceed this range due to an increase in ROS production either as a result of internal physiological changes or external induction. If the cellular antioxidant potential is high enough, acutely increased ROS levels can be quickly reduced again back to the initial (control) range. But if the existing antioxidant potential is not capable of eliminating extra ROS, the cell can increase its antioxidant defenses, but it will require some time to respond, and this will also consume energy and important biomolecules (e.g., amino acids). Upregulation of the antioxidant potential may result in the restoration of ROS levels back into the initial range, or due to a prolonged increase in ROS levels the cell may enter a state of “chronic oxidative stress” (Figure 4). In many cases, acute oxidative stress has no serious consequences for organisms, but the chronic state may lead to or accompany certain pathologies. Oxidative stress is well-documented to occur, for example, in cardiovascular and neurodegenerative diseases, diabetes mellitus, cancer, and aging [9, 12, 47, 51, 86–89]. Under some circumstances, ROS levels do not return to the initial range and the system may be stabilized at new, higher ROS level referred to as “quasistationary” that occurs in various pathological states [81]. Interestingly, the opposite situation of decreased ROS levels can occur in some instances and is sometimes called “reductive stress.” However, there has been very little investigation of this situation and, therefore, it will not be further discussed here.

 The above short excursion into oxidative stress theory underscores not only the importance of GSH for ROS combating in unstressed conditions, but also the augmented role that GSH must play during oxidative stress. Enhanced ROS levels may require not only enhanced GSH action to maintain redox status, but also enhanced energy and material consumption to replace consumed GSH and/or transport it to the places where it is needed.

As mentioned above, GSH may be involved in detoxification of RNS [6]. For example, nitric oxide (^∙^NO) was initially thought to interact directly with GSH to produce *S*-nitrosoglutathione (GSNO). However, further investigation demonstrated ^∙^NO must first be converted to NO^+^ (nitrosonium ion) in an iron- or copper-catalyzed reaction before reacting with GSH to form GSNO [90, 91]. It should be noted that GSNO and other nitrosothiols can be used for storage and transportation of ^∙^NO because as unstable compounds they can be decomposed easily to generate^∙^NO and GSSG.

### 4.2. Elimination of Endogenously Produced Toxicants

The role of GSH in detoxification of the end products of lipid peroxidation such as malonedialdehyde and 4-hydroxy-2-nonenal was mentioned above. Many other toxic metabolites are produced as side-products of the normal cellular metabolism. For example, methylglyoxal (2-oxopropanal) is one of these and it can be generated both enzymatically and nonenzymatically [92, 93]. Glycolysis appears to be the main source of methylglyoxal where it is produced from triose phosphates, particularly due to spontaneous decomposition of glyceraldehyde-3-phosphate [94, 95]. Methylglyoxal toxicity is based on its capacity to interact with any molecule containing free amino groups such as amino acids, nucleotide bases of nucleic acids, and cysteine residues in proteins [96–99]. Methylglyoxal and other *α*-dicarbonyls, in turn, may be involved in ROS generation. Glutathione acts as a cofactor in the system of methylglyoxal elimination which consists of two enzymes called glyoxalases [92, 100, 101]. The first enzyme in this pathway, glyoxalase I (Glo I, EC 4.4.1.5), catalyses the isomerization of hemiacetal adducts, which are formed in a spontaneous reaction between a glutathione and aldehydes such as methylglyoxal:

(9) $$\;\;\begin{array}{cc} \text{glutathione}\;+\;\text{methylglyoxal}\; & ⟷\text{hemithioacetal}\;\text{adduct}\\ & ⟷\text{(R)-S-lactoylglutathione} \end{array}$$

The second enzyme, glyoxalase II (Glo II, EC 3.1.2.6), catalyzes the hydrolysis of the product of the above reaction:

(10) $$\left(\text{R}\right)\text{-}S\text{-Lactoyl-GSH}\;+\;{\text{H}}_{2}\text{O}\;\to \;\text{D}\left(\text{-}\right)\text{lactic}\;\text{acid}\;+\;\text{GSH}$$

This pathway is the main route for methylglyoxal catabolism in yeasts [3, 95, 102, 103] and mammals [104–107].

GSH also may be involved in the detoxification of endogenously produced formaldehyde. For example, some yeasts produce formaldehyde as part of methanol catabolism [108–111]. The reaction is catalyzed by formaldehyde dehydrogenase (FaDH, EC 1.1.1.1) which uses GSH as a cosubstrate:

(11) $$\begin{array}{cc} \text{Formaldehyde}\;+\;\text{GSH}\;+\;{\text{NAD}}^{+} & \to S\text{-formylglutathione}\\ & \;\;+\;\text{NADH}\;+\;{\text{H}}^{+} \end{array}\;$$

Formaldehyde also may be produced from the catabolism of certain amino acids and, therefore, reaction (11) may be important for its detoxification in animals and plants [112, 113]. Interestingly, formaldehyde dehydrogenase also catalyzes the decomposition of *S*-nitroso-glutathione and it is not limited to yeasts [24], but also found in plants and animals [114–117]. 

### 4.3. Metal Homeostasis

GSH can interact with certain metal ions. It contains six potential coordination sites for metal ion binding such as cysteinyl sulfhydryl, glutamyl amino, glycyl, and glutamyl carboxyl groups, and two peptide bonds. Among these, the sulfhydryl group possesses the highest affinity for metal cations, particularly cadmium, copper, zinc, silver, mercury, arsenic, and lead [118]. The interaction of a metal ion with the GSH sulfhydryl group can be stabilized by coordination with other potential binding sites. The most stable complexes are formed by divalent cations in a 1 : 2 ratio with GSH. The complexes form spontaneously because they are thermodynamically favored and the resulting mercaptides are relatively stable. Several metabolic functions for these metal-GSH complexes have been proposed: (i) they can help in the mobilization and transfer of cations between ligands; (ii) they can serve to transport metal ions across membranes; (iii) they serve as a source of cysteine, playing a central role in metal homeostasis; (iv) they serve as a cofactor for redox reactions yielding metal compounds with different speciation or biochemical forms [118]. The remainder of this section focuses only on GSH involvement in the metabolism of chromium, copper, and iron ions.

GSH is involved in Cr^6+^ reduction in many organisms (reviewed in [82, 119]). GSH-dependent reduction of Cr^6+^ results in the formation of Cr^3+^, effectively converting the ion from an anionic form (Cr_2_O_7_^2−  ^or CrO_4_^2−^) to a cationic form [82, 120]. Cr^6+^ in its anion form (associated with oxygen) is readily transported into cells via nonspecific anion carriers, but Cr^3+^ as a cation is not so bioavailable and is believed to be less toxic due to its interaction with many cellular ligands [121]. Therefore, Cr^6+^ reduction to Cr^3+^ can be characterized as a way to decrease chromium toxicity [122]. Although Cr^6+^ can be reduced nonenzymatically, studies suggest that in cells GSH and GSH-dependent enzymes, either alone or in concert with ascorbic acid and cysteine, play an important role in these processes [119, 123–126]. For example, the inhibition of GR by carmustine prevented Cr^6+^ reduction in isolated rat hepatocytes [127]. Nontoxic biological effects of chromium are also associated with GSH-related transformation of Cr^6+^. Although it is not clear how this occurs, these effects are related to the ability of chromium to affect carbohydrate metabolism potentiating the effects of insulin [128, 129]. It is worth noting that although Cr^3+^ is thought to be a regulator of carbohydrate metabolism, the capacity of biological systems to reduce Cr^6+^ with the participation of the GSH system may be used to deliver chromium into biological systems.

GSH plays a more specific and well-documented role in the metabolism of copper and iron. GSH is believed to be responsible for the mobilization and delivery of copper ions for the biosynthesis of copper-containing proteins [118]. In this case, GSH is involved in (i) reduction of Cu^2+^ to Cu^+^, (ii) mobilization of copper ions from stores, and (iii) delivery of copper ions during the formation of “mature” proteins. For the last function, Cu^2+^ must be reduced to Cu^+^ before it can be incorporated into apoproteins, and GSH provides the reducing power [130]. Interestingly, GSH is not only the carrier for Cu^+^, but is also involved in copper mobilization from metallothioneins in a reversible manner. The Cu(I)-GSH complex is used for copper incorporation into Cu,Zn-superoxide dismutase (Cu,Zn-SOD) from bovine erythrocytes [131] lobster apohemocyanin [132], and blood plasma albumin [133].

The role of GSH in iron metabolism is not as well studied. However, by analogy with copper, GSH may be involved in iron reduction, transportation, mobilization from different stores, and incorporation into certain target molecules. GSH involvement in iron metabolism in the yeast *S. cerevisiae,* has been investigated in details [134]. GSH was not required for iron adsorption, delivery to mitochondria, maintenance of mitochondrial Fe,S-proteins, or for their maturation. However, the maturation of extramitochondrial Fe,S-proteins required GSH. Although the precise role of GSH in this process is not clear, GSH involvement in facilitated transport of components of Fe,S-clusters was suggested [134].

## 5. Glutathione Peroxidases and Transferases and Their Regulation

These enzymes play very specific roles in cellular metabolism that should be specially highlighted. As mentioned above, GPx catalyzes the GSH-dependent reduction of many peroxides (reaction (6)). GPx enzymes are particularly involved in the removal of LOOH, thereby terminating lipid peroxidation chain reactions and protecting biological membranes. Four isoenzymes of GPx have been identified in mammalian tissues [135, 136]. The active site of these enzymes contains a selenocysteine residue which is responsible for the catalytic activity. Mammalian isoenzymes GPx-1, GPx-2, and GPx-3 reduce H_2_O_2_ and peroxides of free fatty acids, whereas GPx-4 reduces peroxides of phospholipids and cholesterol [137].

Certain glutathione *S*-transferases (GST, EC 2.5.1.18) catalyze GSH conjugation with electrophiles, but some also catalyze the reduction of lipid peroxides and as a consequence they are also called selenium-independent peroxidases [138]. These GSTs do not possess a selenocysteine residue in their active site. GSTs are an enzyme superfamily responsible for biotransformation of electrophilic compounds. In this way GSTs protect organisms against genotoxic and carcinogenic compounds of both exogenous (xenobiotics) and endogenous origin. Mammalian GSTs are organized in multiple classes designed by Greek letters. Major classes include Alpha, Mu, Pi, abbreviated in Roman capitals as A, M, P. [139]. Traditionally, GST activity is measured with 1-chloro-2, 4-dinitrobenzene (CDNB), cumene hydroperoxide, or *tert*-butyl hydroperoxide as the substrates. Due to selenium-independent GPx activity, *α*-class GSTs can efficiently reduce peroxides of free fatty acids and phospholipids, as well as cholesterol hydroperoxides [140]. It is worth noting that *α*-class GSTs can reduce peroxides of membrane phospholipids without requiring phospholipase A_2_-mediated release of the peroxidized fatty acids from the membrane phospholipids [141, 142]. The role of *α*-GST in peroxide metabolism is highlighted in excellent reviews of Awasthi and colleagues [140, 143].

By regulating the level of certain electrophiles, GSTs and GSH may indirectly affect regulatory pathways controlled by these compounds. For example, 4-hydroxynonenal (4-HNE) is a well-known product of lipid peroxidation, which has a key role in stress-mediated signalling. Its steady-state intracellular level is determined by the balance between production due to lipid peroxidation and elimination via various pathways. One of the subgroups of the anionic *α*-class of GSTs can utilize 4-HNE as a preferred substrate, conjugating it to GSH with high efficiency [140]. The enzyme shows a much higher affinity toward 4-HNE than to most xenobiotics suggesting its critical role in the regulation of cellular 4-HNE levels. The adduct formed, GS-HNE, is exported from cells in an ATP-dependent manner by a primary transport system similar to the system that extrudes other GSH conjugates [144, 145].

However, GSTs may not only play positive roles in cell protection against xenobiotics. In certain cases, they can be responsible for the need to increase the doses of specific drugs. For example, in many solid tumors enhanced resistance to drugs is associated with the increased activity of GSTs that detoxify xenobiotics [27, 146]. GST was identified as a prominent protein in many cases and is overexpressed in many cancers resistant to several drugs. These GSTs have been proven to be a viable target for prodrug activation with at least one candidate in late-stage clinical development [146].

**Figure 5:**
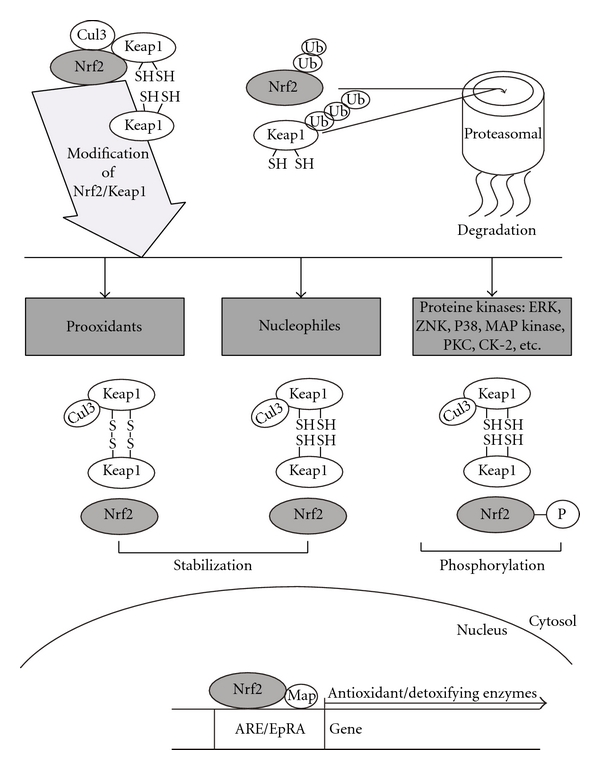
Operation of the Nrf2/Keap1 system during response to oxidative stress in animals. Under nonstressed conditions the transcription factor Nrf2 binds to the Keap1 homodimer. The resulting protein complex can then further complex with Cullin 3 leading to ubiquitination of Nrf2 followed by proteasomal degradation. Following an oxidative insult or electrophilic attack, Keap1 cannot bind Nrf2 which allows Nrf2 to diffuse into the nucleus and, in concert with small Maf proteins (sMaf), Map and others, Nrf2 binds to the ARE/EpRE elements of regulatory regions in genes encoding antioxidant or phase 2 detoxification enzymes. Nrf2 migration into the nucleus is promoted by at least three different mechanisms: oxidation of Keap thiol groups to form disulfides, modification of Keap1 cysteine residues by electrophiles, or phosphorylation of Nrf2 by protein kinases that, in turn, may be activated by oxidants.

The activities of GPxs and GSTs, like other antioxidant enzymes, are regulated in many ways. Most attention has been paid to their upregulation via specific regulatory pathways involving ROS or electrophiles at certain stages. Many reviews extensively describe these pathways [147–152], and here we will describe just a few of them where GSH is known to be an active participant. OxyR-related regulatory protein was described in bacteria about 20 years ago (reviewed in [153–158]). Subsequently, the YAP1/GPx3-regulated system was found to be responsible for augmentation of antioxidant potential in yeast [85, 154, 157, 158]. Finally, in animals the operation of ROS-based regulatory cascades, involving GSH and GSH-related enzymes, has been identified. In this context, the Nrf2/Keap1 system of animals is often considered to be the most important and finely controlled pathway that regulates the activities of antioxidant and phase II detoxification enzymes via interaction with antioxidant response elements (ARE) in regulatory regions of many of the genes that encode antioxidant enzymes [21, 159–168] (the same gene region is also known as the electrophile response element (EpRE) to designate its involvement in the cellular response to electrophiles). In animals, the activities of many phase II detoxifying enzymes, including GSTs and GPxs, are also upregulated via the Nrf2/Keap1 system. The dilemma of the simultaneous regulation of GSTs and antioxidant enzymes was solved when the mechanism by which the Nrf2/Keap1 system operation was uncovered (Figure 5). Under normal (nonstressed) conditions Nrf2 protein interacts with Keap1 in the cytosol and is quickly ubiquitinated followed by the proteasomal degradation. However, when ROS levels rise, Keap1 is oxidized and becomes incapable of binding Nrf2. This results in its migration (possibly related to phosphorylation by certain protein kinases) into the nucleus. In the nucleus, Nrf2 binds to the ARE (EpRE) DNA element of target genes together with a small Maf protein and perhaps with other proteins. The complex stimulates the expression of target genes, including those encoding GSTs and antioxidant enzymes. Clearly, enhanced expression of antioxidants and phase II detoxification enzymes is an important factor in increasing cellular resistance to xenobiotics. In addition to GSTs, a key enzyme of GSH-biosynthesis, *γ*GLCL, is also among the targets of the Nrf2/Keap1 regulatory pathway. Because of its involvement in the regulation of diverse physiological processes, and especially those related to GSH, the Nrf2/Keap1 system has gained attention not only at the basic biological level, but also from a pharmacological viewpoint.

Detoxification of xenobiotics in animals is usually, but not always, provided by a specific system consisting of so-called phase I, phase II, and phase III enzymes. Phase I enzymes are represented by hydroxylases such as endoplasmic reticulum members of the cytochrome P450 family, which introduce oxygen onto molecules of hydrophobic xenobiotics and endogenous compounds, transforming them in more hydrophilic forms. Phase II detoxification enzymes catalyze conjugation reactions that add glutathione, amino acids, sulphate, glucuronic, acetyl, or methyl residues to activated xenobiotics. Plasma membrane antiporters represent phase III detoxification; these energy-dependent pumps export conjugates from the cell, thereby decreasing their intracellular concentration. Although this system of nomenclature for the detoxification of xenobiotics can be useful, the classification may not always hold for detoxification reactions involving GSH. For example, many electrophilic xenobiotics can react directly with GSH without the prior need for activation by phase I enzymes [34].

## 6. Glutathionylation of Cellular Sulfhydryls

**Figure 6:**
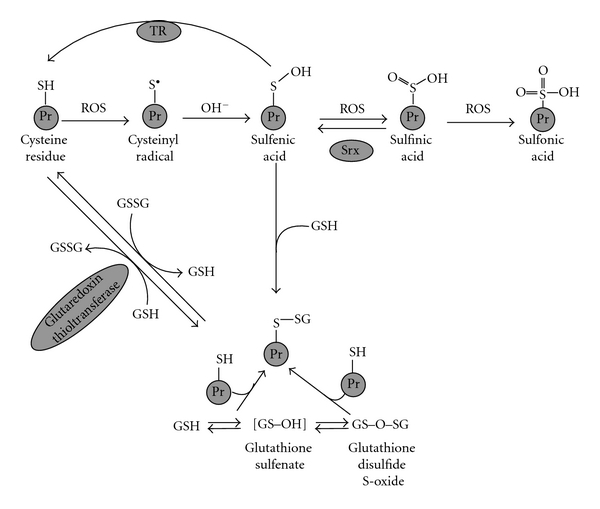
Oxidation of protein cysteine residues to sulfenic, sulfinic, or sulfonic derivatives and formation of glutathionylated proteins. In biological systems, sulfenic and sulfinic derivatives may be reduced by thioredoxin (TR) and sulfiredoxin (Srx), respectively, whereas sulfonic moieties cannot be reduced. Glutathionylated proteins are formed by direct interaction of GSH with sulfenic acid derivatives, exchange between cysteine residues and GSSG, or interaction with oxidized glutathione forms.

An increase in cellular levels of mixed disulfides formed between GSH and protein thiols, a process called glutathionylation, was demonstrated to be caused by oxidative stress about three decades ago [169–171]. Since that time many studies of the role of glutathionylation have been carried out. Work from the laboratory of Sies and others implicated the process in the regulation of the activity of specific enzymes and certain regulatory pathways [6, 172–176]. From this, glutathionylation was recognised as one of the physiologically relevant mechanisms of posttranslational modification of certain proteins. Exposure of cysteine residues of proteins to ROS leads to their oxidation with the consequent formation of stable sulfenic, sulfinic, or sulfonic acid derivatives and unstable transient forms (Figure 6). Sulfenic acid may be returned to the original cysteine form by several reductases ([6, 177] and cited therein) whereas sulfinic acid can be reduced only by the specific action of sulfiredoxin [178–181]. It is believed that sulfonic acid cannot be reduced in living organisms. Cysteine oxidation to sulfenic acid may be used for ROS sensing and in this case it plays a positive role in cell adaptation. However, more frequently the oxidation may inhibit certain proteins if the oxidized cysteine residues are important for protein function. Therefore, in addition to direct reduction of sulfenic acid to cysteine, living organisms possess other ways of dealing with this moiety (Figure 6). Sulfenic acid residues may interact with reduced glutathione forming mixed disulfides [182, 183]. This issue is not so straightforward, because formation of this dithiol can be implicated in the regulation of some metabolic pathways. Many proteins are subject to glutathionylation and some of them lose biological activity as the result, whereas others may be activated [182]. In human T lymphocytes, Fratelli and colleagues [184] found that cell exposure to oxidants (diamide and H_2_O_2_) enhanced glutathionylation of certain proteins. These included cytoskeletal proteins (vimentin, myosin, tropomyosin, cofilin, profilin, and actin), metabolic enzymes (enolase, aldolase, 6-phosphoglucolactonase, adenylate kinase, ubiquitin-conjugating enzyme, phosphoglycerate kinase, triose phosphate isomerase and pyrophosphatase), redox enzymes (peroxiredoxin 1, protein disulfide isomerase, and cytochrome c oxidase), cyclophilin, stress proteins (HSP70 and HSP60), nucleophosmin, transgelin, galectin, and fatty acid binding protein. *S*-Glutathionylation is thought to be one of the mechanisms preventing ROS-induced irreversible protein inactivation under oxidative stress insults. During recovery, GSH residues can be removed from the glutathionylated proteins resulting in restoration of their functional activity.

Figure 6 shows the known pathways of glutathionylation/deglutathionylation and oxidation of cysteine residues in cellular processes. The routes leading to the formation of mixed disulfides are interactions between: (i) sulfenic acid derivatives and GSH, (ii) GSSG and protein cysteine residues, (iii) protein cysteine residues and glutathione sulfenate, and, finally (iv) protein cysteine residues and glutathione disulfide S-oxide. Connected to the protein via a disulfide bond, GSH can be removed via a thiol-disulfide exchange reaction. GSH is used by glutaredoxin releasing GSSG. It is now clear that glutathionylation as a posttranslational modification of proteins can be involved in the regulation of the activity of diverse proteins.

In addition to formation of mixed thiols with proteins, GSH may also form mixed disulfides with low molecular mass thiols. In many cases, the biological relevance is uncertain, but in the case of coenzyme A the formation of the mixed disulfide may be biologically important. For example, CoASSG was found to inhibit GR [185], phosphofructokinase [186], and fatty acid synthase [187], whereas fructose-1,6-bisphosphatase was activated by CoASSG [188]. A very potent vasoconstrictory effect of CoASSG has also been described [189].

GSTs also play regulatory roles in many cellular processes in ways that are not usually directly related to their catalytic activity. Frequently their direct interaction with certain regulatory enzymes/proteins has been shown to be involved in cellular responses to oxidative stress, regulation of proliferation, differentiation, and apoptosis. Most information on these mechanisms is associated with the pi-type GSTs (GST*π*). For example, GST*π* inhibits c-Jun aminoterminal kinase (JNK) [183]. JNK phosphorylation activates c-Jun and triggers activation of multiple downstream effectors related to proapoptotic signalling and certain cytotoxicities but its sequestration in a complex with GST*π* blocks these events. Under oxidative or nitrosative stresses the above complex dissociates, and GST*π* undergoes glutathionylation with subsequent oligomerization. The GST*π* isoenzyme is believed to be the main isoenzyme involved in this effect, although other soluble isoforms of GST may also be involved in this type of regulation [183].

The glutathionylation process is thought to be responsible for the anticancer effect of PABA/NO [*O* ^2^-{2,4-dinitro-5-[4-(*N*-methylamino)benzoyloxy]phenyl}1-(*N*,*N*-dimethylamino)diazen-1-ium-1,2-diolate] [190]. Overexpression of GST*π* in solid tumors is linked to the development of resistance to a number of anticancer agents. PABA/NO is catalytically activated by GST*π* releasing ^∙^NO that elicits antitumor activity both *in vitro* and *in vivo* [191]. Locally produced ^∙^NO extensively modifies specific target proteins, particularly protein disulfide isomerase (PDI). Nitrosylation or glutathionylation of PDI leads to enzyme inactivation, activation of the unfolded protein response (UPR), and cancer cell death. It has been suggested that ^∙^NO itself may not be directly responsible for the toxicity of PABA/NO, but rather that peroxynitrite, which is much more reactive, provides the effect. Peroxynitrite is a product of the interaction between nitric oxide and superoxide anion radical and is known to be a powerful nitrosating agent [190].

## 7. Regulation of Transcription of GSH-Related Genes

Being an important antioxidant either directly, or via GSH-related enzymes, GSH is a key component in the regulation of redox homeostasis. It is well known that changes in GSH levels or deregulation of the redox status are caused or at least are associated with diverse pathologies and aging. The most thoroughly investigated cases include cardiovascular and neurodegenerative diseases, cancer, AIDS, cystic fibrosis, liver disorders, diabetes mellitus, and associated complications. Regulation of the activities of GSH-related enzymes is often considered as a way to prevent or ameliorate the disease. Several cellular signalling systems are known to be involved. However, the mostly efficient approaches are related to the possibility of manipulating GSH biosynthesis and phase II detoxification enzymes. In the former case, attention is focused on the first key enzyme of GSH synthesis, *γ*GLCL, and in the latter case on GSTs. These enzymes are mainly regulated at the expression level and some of the mechanisms involved have been deciphered. Although it is known that the promoter regions of the genes encoding *γ*GLCL and GSTs possess binding sites for such transcriptional regulators as NF-*κ*B, AP-1, AP-2, SP-1, and others [192–194], most attention has been concentrated on the Nrf2/Keap1 system [160, 195]. This is connected, at least partially, to its high sensitivity to effectors relative to other regulatory systems [81]. The Nrf2/Keap1 system is responsive to many challenges, particularly to oxidants and electrophiles. As mentioned above, Nrf2 operates in concert with an adaptor protein, Keap1, a cytoplasmic resident. In nonstressed cells the binding of Nrf2 to Keap1 promotes ubiquitination of Nrf2 followed by proteasomal degradation. This system is tightly regulated in cells (Figure 5). Enhanced levels of oxidants or electrophiles, as well as activation of various protein kinases disrupt the Nrf2/Keap1 association resulting in Nrf2 stabilization and migration into the nucleus. Therein Nrf2 binds to the ARE/EpRE in the promoter region of target genes and in concert with small proteins of the Maf family stimulates their transcription. In a series of elegant studies several mechanisms that direct Nrf2 into the nucleus have been described (reviewed in [81]): (i) oxidation of specific cysteine residues of Keap1 resulting in its inability to bind Nrf2, (ii) interaction of nucleophilic molecules with cysteine residue(s) of Keap1 leading to the formation of adducts that prevent binding to Nrf2, (iii) phosphorylation of Nrf2 by different protein kinases, and (iv) ubiquitination of Keap1 followed by proteasomal hydrolysis (Figure 5).

Deciphering the mechanisms of operation of the Nrf2/Keap1 system helped to explain various previously puzzling data on chemoprevention in several disease states. Chemoprevention has attracted much attention as one of the most practical and realistic strategies for decreasing the global burden of diseases related to xenobiotics and certain oxidants. A mechanistic approach has gained acceptance recently because it not only provides the rationale to reveal potential mechanisms, but it also predicts and identifies potentially effective chemicals. A broad spectrum of substances have been reported that exhibit chemopreventive potential, and it is noticeable that many of these substances were identified in plants, particularly those that are medicinal and/or edible. Numerous phytochemicals derived from fruits, vegetables, grains, spices, and herbs are capable of affecting certain diseases related to disrupted GSH homeostasis. Extensive reviews on chemopreventive phytochemicals have been published. Thus, there is no need for in depth coverage of this field, and interested readers are directed instead to several excellent recent reviews [21, 22, 86, 166, 195–198]. In the present review, discussion will be limited to well-studied phytochemicals that operate by affecting the Nrf2/Keap1 system. These have been exceptionally well discussed by Surh and colleagues [21] and are summarized in Table 1.

Sulforaphane [1-isothiocyanato-(4*R,S*)(methylsulfinyl)butane] is an isothiocyanate found in broccoli and other cruciferous plants. It is a known inducer of genes encoding phase II defense and antioxidant enzymes including GPx, GST, and *γ*GLCL [196, 211]. Sulforaphane appears to modulate upstream MAP kinases, but reliably demonstrated effects are associated with Nrf2 activation via the direct modification of Keap1 cysteine residue(s) [199]. As an electrophile, sulforaphane directly interacts with protein thiols forming thionoacyl adducts. In addition, sulforaphane induces structural changes in Keap1 leading to its polyubiquitination and proteasomal degradation [200]. 

Curcumin (diferuloylmethane) is derived from the rhizomes of turmeric (*Curcuma longo*). It stimulates the expression of antioxidant and phase II detoxification enzyme genes in several experimental models [212–214]. Curcumin-induced expression is also mediated via Nrf2 activation in a ROS-related manner. ROS activate PKC and P38 MAP kinase which then have downstream effects by phosphorylation of Nrf2 [201, 215]. 

Epigallocatechin gallate (EGCG) is a major active catechin of green tea that exerts antioxidant, anti-inflammatory and chemopreventive properties [86, 216, 217]. It stimulates Akt, ERK1/2 and P38 MAP kinase leading to Nrf2 phosphorylation and its import into the nucleus [202, 218]. 

Several allyl sulfides, namely, diallyl sulfide (DAS), diallyl disulfide (DADS), and diallyl trisulfide (DATS) are major components of garlic that are capable of inducing phase II detoxification enzymes in a Nrf2-dependent manner [203, 204, 219]. DAS transiently increases ROS concentrations stimulating, ERK and P38 MAP kinase which phosphorylate Nrf2 [203, 220]. 

Resveratrol (trans-3,5,4′-trihydroxystilbene) is a polyphenol found in grapes, bilberry, blueberry, other berries, and other plant species. It exerts antioxidant, anti-inflammatory, antiaging, and chemopreventive activities affecting cellular signalling [205, 221, 222]. These activities are mediated, at least partially, by Nrf2 phosphorylation. 

Pungent vanilloids such as capsaicin (trans-8-methyl-N-vanillyl-6-nonenamide), a major pungent of hot chili pepper (*Capsicum annuum*) [206, 223], and (10)-shogaol from ginger (*Zingiber officinale*) also activate phase II detoxification enzyme expression in a Nrf2-dependent manner [207]. The former acts in a ROS-dependent manner via PI3/Akt mediated Nrf2 phosphorylation, whereas the latter acts via electrophilic alkylation of Keap1. 

Lycopene, a natural carotenoid found in tomato and tomato products also exerts chemopreventive activity in an Nrf2-dependent manner [208, 224]. However, there is no available information on the mechanisms involved. It should be noted, that absorption of lycopene by the intestine is much more efficient from processed tomatoes than from fresh tomatoes due to a higher bioavailability in the processed products [225–227]. 

Carnosol, an orthophenolic diterpene found in rosemary (*Rosmarinus officinalis*), also enhances the expression of phase II detoxification enzyme genes in an Nrf2-related manner [228]. Upregulation of ERK, P38 MAP kinase, and JNK pathways was found to be responsible for the effects, which potentially show the involvement of Nrf2 phosphorylation [228]. Cinnamaldehyde from dried bark of *Cinnamomum cassia* also induced phase II enzyme expression via Nrf2 translocation into the nucleus [209, 229, 230]. Xanthohumol, a sesquiterpene from hop (*Humulus lupulus*) also shows chemopreventive activity, inducing antioxidant and phase II detoxification enzymes [210]. Its action was linked with Nrf2 activation resulting from the alkylation of Keap1. Hence, a great variety of diverse agents of natural origin have been found that activate the Nrf2 signalling pathway, but it is likely that many more remain to be discovered. 

Many diverse studies on the involvement of Nrf2 and associated components were discussed above. However, in our opinion, the authors have not always provided clear evidence of direct or mediated Nrf2 involvement in the upregulation in certain systems. Although Nrf2 involvement could be expected logically, other signalling pathways should also be investigated. This is especially true when dealing with natural extracts instead of pure compounds because even a minor component in the extract may affect the system via an unidentified pathway(s) and imitate Nrf2 involvement. Unfortunately, in many cases the data presented do not provide definitive evidence to support the involvement of Nrf2. 

The chemopreventive efficacy of various phytochemicals that has been demonstrated in cell models frequently cannot be extrapolated to whole organisms due to low bioavailability. Only a very small portion of consumed phytochemicals is absorbed in the gastrointestinal tract, usually much less than 1% [231, 232]. In addition, there are often potentially negative effects on organisms due to supposedly useful phytochemicals. They often activate the expression of genes encoding phase I detoxification enzymes such as cytochrome P450. This can create problems because many xenobiotics may be activated by oxidation mediated by these oxygenases and thereby express their toxic potential. In this case, the transcriptional activation of genes encoding these oxygenases would be considered a negative side effect of phytochemical treatment. In some cases, these compounds may simultaneously activate the expression of phase I and phase II enzyme genes, in which case the final result would be unpredictable in many circumstances. Simultaneous induction of the expression of genes encoding phase II detoxification and antioxidant enzymes may take place with so-called phase III detoxification enzymes which are membrane pumps providing active extrusion of GSH conjugates of electrophiles that are formed either spontaneously or enzymatically in GST-catalyzed reactions. A final important issue must be emphasized when analyzing effects due to phytochemicals. Phase II and phase III detoxification enzymes may be responsible for catabolizing certain drugs (such as drugs used to treat cancer) via conjugation with GSH and extrusion from cells. This could lead to the need to increase doses of some drugs to make them effective or could even result in resistance to the drugs. 

The mechanism of induction of phase II enzyme expression by plant polyphenols has been elucidated by Zoete and colleagues [233]. They investigated the ability of these compounds and their synthetic analogs to induce the activity of NADP(H) quinone reductase (NQ01), a prototypic phase II detoxification enzyme. By using quantum-mechanical methods the authors calculated the tendency of these compounds to release electrons by the energy of the highest occupied molecular orbital (E_HOMO_). They found that the smaller the absolute E_HOMO_ of an agent (i.e., the lower its reduction potential), the stronger its electron donor property was and the greater its inducer potency. That allowed inducers to be ranked and led to predictions of the efficiency of inducers based on their reduction potential [233]. However, it should be noted that the experiments were carried out in cell culture, which does not take into account factors such as the absorption and transportation of polyphenols when they are administered to the whole organism. However, the approach may give some clues for the prediction of the biological effects of polyphenols in regulating the activity of antioxidant and phase II and III detoxification enzymes. 

## 8. Relationship between GSH Homeostasis and Pathologies

Elevated ROS levels as well as the presence of different xenobiotics are well-known factors in various pathologies and aging, but in some cases these relationships are not straightforward. Many details of GSH involvement in these processes including regulation of GSH-related enzymes were discussed above. Therefore, the current section will provide a general summary as well as highlight some potentially useful therapeutic avenues. 

**Figure 7:**
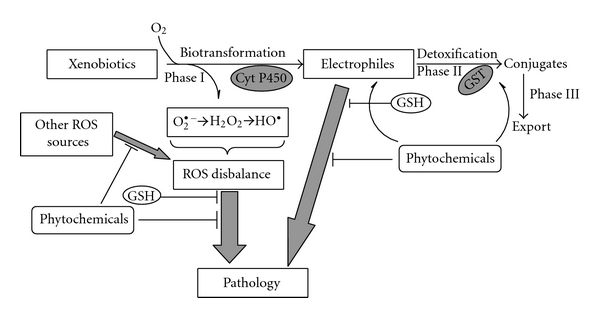
Involvement of glutathione in the detoxification of xenobiotics and reactive oxygen species, its relationship with pathological development and the potential role of different phytochemicals. Glutathione is responsible for helping to maintain redox balance by directly or indirectly interacting with ROS, and is also involved in detoxification of electrophiles either via direct interactions or via enzyme-catalysed conjugation. Certain phytochemicals may affect GSH action on ROS and electrophiles either by directly interacting with ROS and electrophiles, or by upregulating defensive enzymes.

Figure 7 shows general routes of enhanced ROS levels and/or the presence of xenobiotics associated with various pathologies. Elevated ROS levels are a key finding in many diseases [234] including cardiovascular and neurodegenerative diseases, cancer, diabetes mellitus, and aging [88, 89, 197, 235–237]. ROS concentration may be enhanced for many reasons of both an internal or external nature, such as inflammation or exposure to xenobiotics. GSH can interact directly with ROS to reduce their levels and in this manner delay the development of pathologies. The potential of various phytochemicals to disrupt this link between ROS elevation and increased pathology may be related to the inherent antioxidant activity possessed by various plant components. However, potentially more potent protective effects of phytochemicals may arise from indirect effects. Since this review is focused on GSH, the ways in which GSH participates in these processes must be highlighted. They include (1) activation of GSH biosynthesis via supplementation of substrates and energy, (2) increased enzymatic potential to produce GSH and reduce GSSG, (3) increased activities of detoxification enzymes that use GSH, and (4) activation of routes for extrusion of GSSG and glutathione *S*-conjugates from cells. It is clear from this list that there are several good targets for pharmacological interventions in pathologies in which oxidative stress may be a contributing factor.

The uptake of xenobiotics and their interaction with biomolecules in living organisms depend on various factors such as their chemical and physical properties, type of organism, and its physiological state. Here, we will not focus on specific aspects, but rather will provide the general principles of xenobiotic metabolism leading to pathologies, GSH involvement and potential protective effects of certain phytochemicals. Some xenobiotics can be directly autoxidized leading to ROS production and the potential pathological consequences were described above. However, most xenobiotics are not autoxidized directly and contribute to pathology only after transformation via different mechanisms. Many xenobiotics are oxidized by various endogenous oxygenases with the production of ROS at this stage. The biotransformed xenobiotics that result may also have enhanced potential to induce pathology via direct interaction with cellular constituents due to their electrophilic nature. Biotransformed xenobiotics may also undergo autoxidation with concomitant ROS generation. In order to prevent this scenario, cells utilize phase II detoxification enzymes. GSH plays a prominent role in this process, either directly conjugating with xenobiotics or participating as a substrate in enzymatically catalyzed conjugation reactions. Finally, conjugates are excreted from the cell by the phase III detoxification system of plasma membrane active transporters. However, cellular GSH is not lost to a great extent; most is reclaimed via GSH salvage processes (Figure 2). This means that extracellular transpeptidases cleave the conjugates releasing different GSH components which may be reabsorbed by cells and reused for tripeptide resynthesis. Overall, then, GSH may prevent the development of pathology related to electrophiles either by directly interacting with them or in an enzyme-catalyzed manner. Some phytochemicals also directly interact with electrophiles, but their action may also be realized through activation of GSH synthesis/resynthesis and reduction. Activation of phase II and III detoxification enzymes is thought to be the main route for xenobiotic detoxification and excretion from the organism. Activation of the transcription of genes encoding enzymes that combat xenobiotics is one of the main pharmacological strategies for treating xenobiotic-induced diseases. As described above, the Nrf2/Keap1 system, in concert with other signal transduction systems, regulates the expression of genes encoding many of the enzymes involved in phase I, II, and phase III xenobiotic detoxification. Some phytochemicals may stimulate phase I detoxification enzymes and also increase cellular potential for detoxification of drugs, which may cause either a decrease in sensitivity to the drug or even complete resistance. This emphasizes the need for a clear understanding of the molecular mechanisms of both drug and phytochemical action for the development of new medical strategies.

## 9. Research Tools and Pharmacological Approaches to Manipulate Glutathione Levels

The role of GSH in the function of living organisms is clearly reflected by a phrase coined by Sies [4]—the term “inevitable GSH.” The great importance of GSH has been revealed in multiple experiments either by depletion or repletion of cellular GSH reserves. 

Cellular GSH reserves can be depleted in at least three different ways—by increasing GSH oxidation, by inhibition of biosynthesis, or by inactivation of the genes encoding the enzymes of GSH synthesis. Experimentally, the cellular GSH pool can be reduced by treatment with different oxidants such as hydrogen peroxide (H_2_O_2_), *tert*-butyl hydroperoxide [238, 239], or diamide [240, 241]. In 1979, a specific inhibitor of *γ*GLCL was synthesized—buthionine sulfoximine (BSO) [13, 14], that when introduced into cells depletes GSH reserves [16, 17, 242]. These approaches have helped researchers investigate the function of cellular GSH. Since the use of oxidants to deplete GSH pools in the treatment of different pathologies usually causes many side effects, BSO was soon tested not only for basic research purposes, but also for clinical investigations in cancer research. For example, local BSO application to certain skin cancers may sensitize them to irradiation [243], drug [244], and photodynamic [245] treatments. 

Frequently used tools in GSH research and therapy are interventions that increase GSH levels [246]. This is usually achieved by supplementation with GSH monoesters and diesters [247–249], GSH precursors such *N*-acetyl cysteine (NAC); [250–252] or *α*-mercaptopropionylglycine [87, 253]. Importantly, cysteine is not usually utilized as a precursor presumably due to its toxicity at high concentrations [254]. On the other hand, cysteine in protein-bound form, particularly as a component of whey, has some potential to increase GSH levels [255–258]. The above compounds are used as precursors for GSH biosynthesis, both experimentally and in some therapies; for example, NAC is broadly used in therapies that combat HIV [259–261] and other infections [262–264]. Although used less frequently than NAC, cysteine precursor in the form of prodrug, 2-oxothiazolidine-4-carboxylate (OTC), is also used to enhance cellular GSH level [247]. Experimentally, the overexpression of certain genes involved in GSH production also may enhance its level. 

At least one important factor needs to be taken into account when treatments are used to elevate GSH. It is well known that many “classic” antioxidants can, under certain conditions, become prooxidants. These include low molecular mass antioxidants such as ascorbic acid [265, 266], epigallocatechin-3-gallate [267], *α*-tocopherol [268], and retinol [265], as well as antioxidant enzymes such as superoxide dismutase [269, 270]. Although information on possible prooxidant properties of GSH is very limited [271–273], its potential prooxidant effects cannot be ignored. Virtually all compounds known as antioxidants possess prooxidant properties [274]; these are two sides of the same coin. The relationship between pro- and antioxidant properties depends on the nature of the compound and specific conditions.

## 10. Conclusions and Perspectives: Glutathione—Two Faced Janus Pharmacological Target

GSH has a very complicated pattern of involvement in diverse biological processes. Consequently, any experimental and clinical intervention should be undertaken with precaution due to the complicated, interrelated, and tightly regulated networking of living processes. In many cases, any modification of one parameter may result in unpredictable responses from diverse processes. For example, at first glance, an increased GSH level through supplementation of its esters may augment defense mechanisms of not only normal cells, but also of cancer cells, especially considering that cancer cells may be rather aggressive in sequestering resources. This can result in a need to enhance the doses of anticancer drugs.

The same ideology can be applied to upregulation of detoxification and antioxidant enzymes. They are frequently regulated at the transcriptional level via enhanced Nrf2 binding to ARE/EpRE DNA elements. However, in many cases, phase II and III detoxification enzymes are also responsible for the detoxification of anticancer drugs and their extrusion from the cell. In addition, some inducers of these enzymes affect phase I detoxification enzymes, which frequently may transform procarcinogens to actual carcinogens via metabolic activation by hydroxylases such as cytochrome P450.

However, taking into account the potential undesirable effects of pharmacological interventions, there is a need to investigate them carefully and many different models may be used for that purpose. Based on available information, some specific molecules with expected properties can be synthesized and tested. Several important notes should be provided in this case. Many potential effectors can exist in several forms and chemical synthesis may lead to the production of, for example, mixtures of different racemates or diastereoisomers, some of which may be pharmaceutically effective, but others of which may cause deleterious effects such as what occurred with thalidomide. One of its racemates was teratogenic [275]. The second important consideration in the chemical synthesis of putative drugs is related to the production of intermediates and side products, which needs special attention and investigation.

Another important factor should be reiterated here. Innumerable studies have shown that GSH is an antioxidant. However, virtually any antioxidant can, under certain conditions, act as a prooxidant [274]. For example, in studies with yeast we found that superoxide dismutase may act either as an anti- or prooxidant depending on its expressed activity [269, 270]. Under certain conditions GSH also can be a prooxidant [276]. Therefore, precaution should be paid to interventions that enhance GSH levels.

Because of the above caveats, modern pharmacology research has refocused on natural products, mainly of plant origin, although bacteria, fungi, and animal sources cannot be ignored. The ideal situation is when these components are possessed by edible vegetables, fruits, herbs, and spices or products formed during their processing. Excellent examples of these include sulforaphane from cruciferous plants [196, 211], epigallocatechin gallate from green tea [86, 216, 217], curcumin from turmeric [201, 212–215], allyl sulfides from garlic [203, 204, 219, 220], anthocyanins and resveratrol from different berries and grapes [196, 205, 221, 222], and carnosol from rosemary [228]. These and other examples demonstrate the great potential for discovery of natural compounds that can be used as pharmaceuticals that may affect GSH homeostasis.

Careful selection of experimental models is very important. Cell cultures are extremely useful for the identification of potential drugs. They allow rapid testing of diverse potential compounds at low cost. This approach is especially helpful for revealing molecular mechanisms of investigated processes. In some cases, simpler cellular models such as bacteria and unicellular yeasts can also be used as models, but in many cases their pathways of xenobiotic catabolism are very different from those of mammals thereby limiting their use. However, all isolated cell systems have at least two serious limitations. The first is that isolated cells are not under systemic control by the whole organism, lacking factors such as the regulatory effects of endocrine and nervous systems, which may substantially modify cellular responses. The second is that chemicals or mixtures for testing are applied directly to cells, which avoids complicated whole organism processes such as absorpton, transportation, transformation, and excretion. These processes can lead to large differences in the responses of isolated cells versus cells in intact organisms, emphasizing the fact that both basic and applied studies must ultimately rely on the use of whole animal models.

Animal models also have some limitations, both technical and ethical. The second is beyond the scope of this review, and, therefore, we will focus only on the first item. First, animal experimental models are much more expensive and require many more resources than cellular models. Certainly, mammalian models are the most valuable because these animals are closest to the human condition. However, much information may be gained from simple animal models that may be ultimately applied to mammals. The fruit fly, *Drosophila melanogaster*, is one of the most popular and tractable animal models. Although it is an invertebrate, it is easy to care for, thousands of different strains exist, and it is possible to manipulate its genome. As a result several experimental models of human pathologies have been developed in *D. melanogaster*, making it a very useful biomedical tool. Many biological processes and their regulation are highly conserved in eukaryotes, particularly from yeasts through insects and to vertebrates. For example, the Nrf2/Keap1 system has recently been described in *D. melanogaster* [277] and fish [278]. Warm-blooded mammals, such as rats, mice, and primates are also extremely useful subjects, but ethical issues often substantially limit the use of mammalian models. As a result, cellular models are often preferred to animal models. Certainly, clinical trials in human populations are the final step before introduction of certain drugs.

One more aspect which is frequently ignored should be highlighted here. This is the problem of accurate measurement of the levels of different glutathione forms, particularly reduced (GSH) and oxidized (GSSG) forms, their ratio (an index of redox potential), and mixed thioethers need further experimental development. This is very important because these parameters are used to characterize the development of oxidative or nitrosative stresses under some circumstances, particularly in certain pathologies [9, 10, 27, 146]. When dealing with cell cultures or unicellular organisms it is practically impossible to isolate cells from the cultivation media and fix GSH level quickly. Other problems exist when studying multicellular organisms. One is the need for very rapid dissection and freezing of target tissues because the redox state in cells can change very rapidly. Another is the fact that many organs consist of multiple cell types which can possess different glutathione levels and forms. In other words, global analysis of the whole tissue may give incorrect assessments of glutathione status in different cell types. Finally, there is an issue of the intracellular distribution of glutathione and its metabolites. Disintegration of the cell to isolate subcellular components may result not only in glutathione redistribution, but alter the redox ratio of reduced to oxidized forms, that is, redox potential. New approaches, particularly to resolve *in vivo* glutathione quantification, are needed to solve these and related problems.

Therefore, a scheme for investigation of potential chemicals, pharmaceuticals, or phytochemicals that target GSH homeostasis may be proposed. At the first stage of investigation, cell cultures and unicellular organisms can be used to identify potential candidate compounds and potential effectors and, if possible, to identify mechanisms involved. Selected compounds would then be evaluated at the whole organism level. Studies with *D. melanogaster* are easy and cheap to perform, and existing or specially produced fly lines with deleted regulatory/effector systems may be tested to provide further clues as to the biological action and side-effects of the candidate compound. Zebrafish (*Danio rerio*) also can be used as an alternative genetically tractable model organism, the genome of which has been sequenced, and many tools for molecular interventions in this organism have been developed. Indeed, there are reliable data on the possibility of manipulating the Nrf2/Keap1 system in these fish [279]. If successful in these organisms, candidate compounds of interest may then be studied in mammalian models. The development of molecular biological tools and production of lines with deleted genes or chimeric lines may also provide some additional information. Research with genetically transformed mice would provide the most useful information, but they are expensive and time consuming to work with. So, the final strategy would depend on many circumstances and rely on the facilities available, particular interests, skills and experience of reserchers.

## Figures and Tables

**Table 1:** Phytochemicals that are known to activate the Nrf2/Keap1 signalling pathway in human and animal systems with identified mechanisms.

Phytochemical	Keap1			Nrf2	References
	Oxidation	Alkylation	Ubiquitination	Phosphorylation	
Sulforaphane	−	+	?	?	[199, 200]
Curcumin	+	−	−	?	[201]
Epigallocatechin gallate				+	[202]
Allyl sulfides	?			+	[203, 204]
Resveratrol				+	[205]
Capsaicin				+	[206]
(10)-Shogaol		+			[207]
Lycopene					[208]
Carnosol				+	[209]
Xanthohumol		+			[210]
